# International Classification of Diseases (ICD)-coded obesity predicts risk of incident osteoporotic fracture

**DOI:** 10.1371/journal.pone.0189168

**Published:** 2017-12-07

**Authors:** Shuman Yang, Lisa M. Lix, Lin Yan, Aynslie M. Hinds, William D. Leslie

**Affiliations:** 1 Department of Epidemiology and Biostatistics, School of Public Health, Jilin University, Changchun, Jilin, China; 2 Department of Community Health Sciences, University of Manitoba, Winnipeg, Manitoba, Canada; 3 Department of Internal Medicine, University of Manitoba, Winnipeg, Manitoba, Canada; University of Nevada Las Vegas, UNITED STATES

## Abstract

International Classification of Diseases (ICD) codes have been used to ascertain individuals who are obese. There has been limited research about the predictive value of ICD-coded obesity for major chronic conditions at the population level. We tested the utility of ICD-coded obesity versus measured obesity for predicting incident major osteoporotic fracture (MOF), after adjusting for covariates (i.e., age and sex). In this historical cohort study (2001–2015), we selected 61,854 individuals aged 50 years and older from the Manitoba Bone Mineral Density Database, Canada. Body mass index (BMI) ≥30 kg/m^2^ was used to define measured obesity. Hospital and physician ICD codes were used to ascertain ICD-coded obesity and incident MOF. Average cohort age was 66.3 years and 90.3% were female. The sensitivity, specificity and positive predictive value for ICD-coded obesity using measured obesity as the reference were 0.11 (95% confidence interval [CI]: 0.10, 0.11), 0.99 (95% CI: 0.99, 0.99) and 0.79 (95% CI: 0.77, 0.81), respectively. ICD-coded obesity (adjusted hazard ratio [HR] 0.83; 95% CI: 0.70, 0.99) and measured obesity (adjusted HR 0.83; 95% CI: 0.78, 0.88) were associated with decreased MOF risk. Although the area under the receiver operating characteristic curve (AUROC) estimates for incident MOF were not significantly different for ICD-coded obesity versus measured obesity (0.648 for ICD-coded obesity versus 0.650 for measured obesity; *P* = 0.056 for AUROC difference), the category-free net reclassification index for ICD-coded obesity versus measured obesity was -0.08 (95% CI: -0.11, -0.06) for predicting incident MOF. ICD-coded obesity predicted incident MOF, though it had low sensitivity and reclassified MOF risk slightly less well than measured obesity.

## Introduction

Obesity is a major public health problem. Worldwide, in 2014, more than 1.9 billion and 600 million adults were overweight and obese, respectively [1].The number of adults with obesity is projected to keep increasing [2]. Obesity leads to greater healthcare expenditures, and higher risk of mortality, and chronic conditions such as type 2 diabetes and cardiovascular disease [3–6].

Despite the health burden attributed to obesity, it is often challenging to capture this information on a population-wide basis. It is impracticable to measure body weight and height for each individual in the population. Therefore, a number of studies have explored the use of diagnosis information captured in population-based administrative healthcare data for ascertaining obesity [7, 8]. Specifically, International Classification of Diseases (ICD) codes have been used to ascertain individuals who are obese. Studies have demonstrated ICD-coded obesity has low sensitivity but high specificity [7, 8]. However, there has been limited research about the predictive value of ICD-coded obesity for major chronic conditions at the population level.

Obesity is generally considered to be protective against osteoporotic (i.e., fragility) fractures partly because individuals with obesity normally have higher bone mineral density (BMD) [9]. Obesity is included in many fracture risk assessment tools (i.e., Fracture Risk Assessment [FRAX] tool [10] and Garvan Fracture Risk Calculator [11]).

For the current research, osteoporotic fractures served as a case study for testing the usefulness of ICD-coded obesity from administrative data for assessing risk of osteoporotic fractures. This research could help to improve fracture risk assessment at the population level, for which data on measured body weight and height are often not available. The aims of this study were to estimate the sensitivity, specificity, positive predictive value (PPV) and negative predictive value (NPV) for ICD-coded obesity where measured obesity was the reference standard, and determine the utility of ICD-coded obesity versus measured obesity for predicting incident major osteoporotic fracture (MOF).

## Materials and methods

### Study setting and participants

The province of Manitoba in Canada has a universal publicly-funded healthcare system, which generates administrative data for the population. These administrative data comprehensively capture hospitalizations, physician visits, and retail pharmacy dispensations [12, 13]. These administrative data have been linked to the Manitoba Bone Mineral Density Database (MBMDD), a population-based clinical registry containing all bone mineral density (BMD) results from dual-energy x-ray absorptiometry (DXA) for the province of Manitoba [10, 14]. Since 2000 the MBMDD contains directly-measured height and weight. The Manitoba Centre for Health Policy (MCHP) at the University of Manitoba maintains anonymized but linkable copies of these data for research purposes. We obtained permission to access these research data from the Manitoba Health Information Privacy Committee. This research was approved by the Health Research Ethics Board of the University of Manitoba; permission for data access was granted by the Manitoba Health Information Privacy Committee.

### Study population

**Fig 1:**
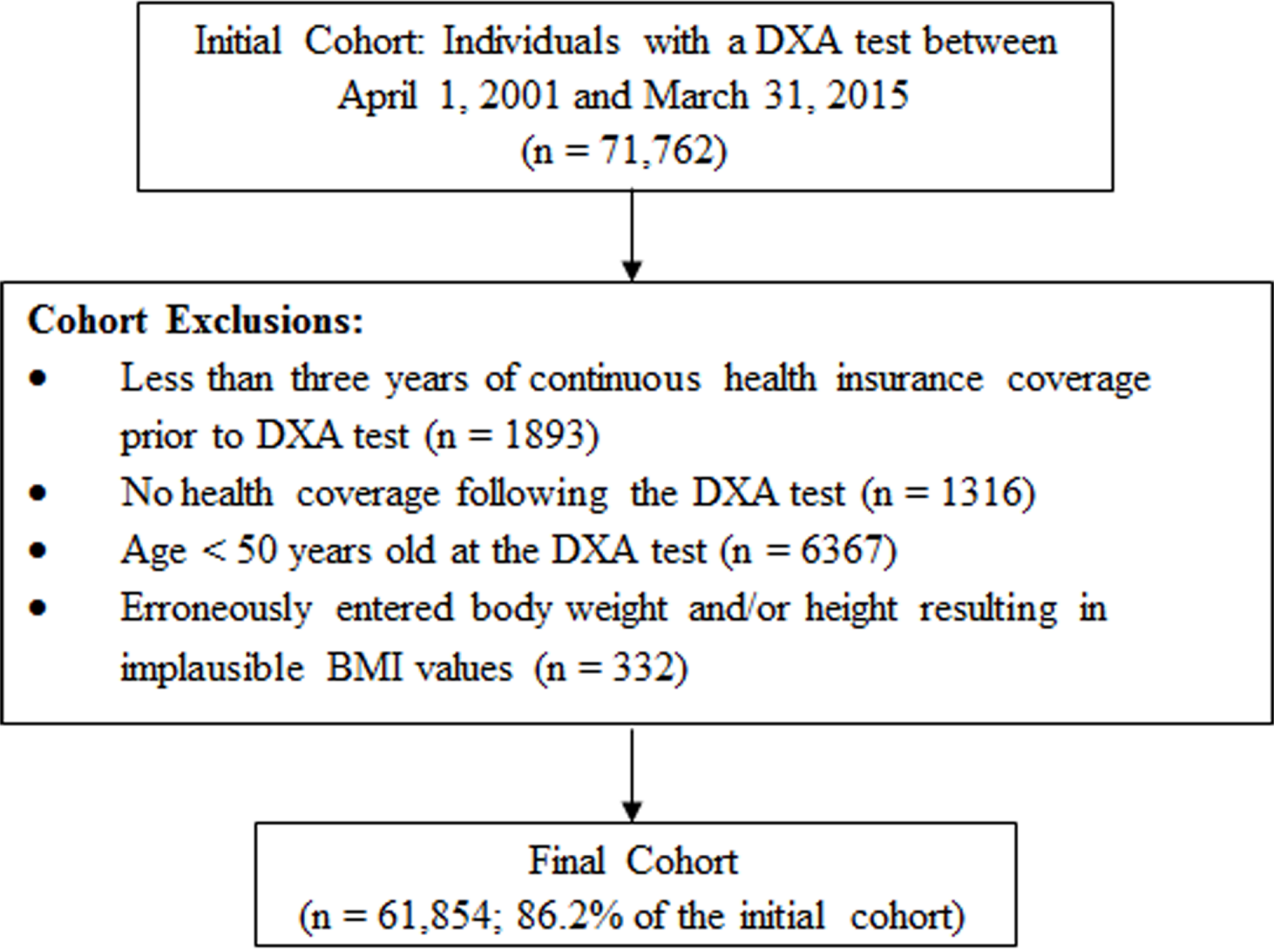
Flow chart for cohort selection and exclusions.

We identified all individuals aged 50 years and older with a DXA test between April 1, 2001 and March 31, 2015 (Fig 1). We excluded all individuals with less than three years of continuous health insurance coverage prior to the DXA test, with no health coverage following the DXA test (minimum requirement 1 day), with age < 50 years old at the time of the DXA test, or with erroneously entered body weight and/or height resulting in implausible BMI values. The purpose of limiting the study to individuals with at least three years of continuous health insurance coverage prior to the DXA test was to collect complete data on the covariates, which are described below.

### Ascertainment of study measures

In Manitoba, hospital discharge abstracts contain diagnoses recorded using the ICD-9-Clinical Modification (ICD-9-CM) coding system prior to 2004 and ICD-10-Canadian Adaptation (ICD-10-CA) coding system from 2004 onwards. For diagnoses coded in physician billing claims, the ICD-9-CM coding system was used throughout the study period. Although there was a coding system change for hospital discharge abstracts, the validity of ICD-9-CM and ICD-10-CA has been shown to be generally similar in recording obesity and other clinical conditions [15]. Individuals with at least one hospital diagnosis code (ICD-9-CM: 278, V85.2-V85.4, and V45.86; ICD-10-CA: E65-E68) or physician diagnosis code (ICD-9-CM: 278, V85.2-V85.4, and V45.86) for obesity within three years prior to the DXA test were defined to have ICD-coded obesity [16, 17]. Measured obesity was defined as BMI ≥ 30 kg/m^2^ [18], and was captured at the time of the DXA test; body weight was measured by a standard scale and body height was measured by a wall-mounted stadiometer. BMI was calculated as body weight (kg) divided by the square of body height (m^2^).

We identified incident MOF (i.e., hip, forearm, clinical spine or humerus fracture) from hospital (the data source for hip fracture diagnosis information) and physician ICD codes (an additional source for forearm, clinical spine or humerus fracture diagnosis information) using validated case definitions [19, 20]. The follow-up was censored at death, out-of-province migration or end of the study period. Any incident fracture associated with high-trauma ICD codes was excluded. MOF rates identified from these case definitions are comparable to those from the population-based Canadian Multicentre Osteoporosis Study (CaMos) [19], which directly confirmed MOF from x-ray and clinical records.

The covariates selected for this study are known MOF risk factors, and include age, sex, femoral neck T-score, prior fractures, prolonged glucocorticoid use, chronic obstructive pulmonary disease (COPD) diagnosis, alcohol/substance abuse diagnosis, rheumatoid arthritis diagnosis, income quintile, and osteoporosis treatment. Femoral neck BMD was measured by DXA (Prodigy and iDXA, GE Healthcare); the reference standard for calculating the femoral neck T-score was based on National Health and Nutrition Examination Survey (NHANES) III database for US White females [21]. Prior fractures were identified within three years prior to the DXA test. Prolonged glucocorticoid use within one year prior to the DXA test (defined as having more than 90 days of use) and osteoporosis treatment (two or more dispensations of bisphosphonate, denosumab, calcitonin, systemic estrogen product, raloxifene or teriparatide) within one year prior to the DXA test or up to one year following the DXA test were captured in the province-wide retail pharmacy database system. We ascertained rheumatoid arthritis, COPD and alcohol/substance abuse diagnoses based on at least one hospitalization or two physician visits with a relevant diagnosis during the three years prior to the DXA test. COPD and alcohol/substance abuse are useful proxies for smoking and high alcohol intake, respectively [22]. Neighborhood-level income (a proxy for socioeconomic status) was extracted from 2006 Statistics Canada population census data and used to classify the cohort into quintiles (lowest income in quintile 1 and highest income in quintile 5) [23].

### Statistical analysis

We described the characteristics of the cohort stratified by ICD-coded obesity and measured obesity using means (standard deviations) for continuous variables and percentages for categorical variables. To address the first objective, sensitivity, specificity, PPV and NPV were estimated for ICD-coded obesity using measured obesity as the reference. We stratified these estimates by osteoporosis treatment and by sex.

To address the second objective, we produced the cumulative incidence curves to demonstrate the association of obesity (ICD-coded obesity and measured obesity) with incident MOF. Log-rank tests were used to assess differences in the cumulative MOF risk between individuals who were and were not classified as obese using ICD-coded obesity and measured obesity. We used unadjusted and adjusted Cox proportional hazards models to test the associations of ICD-coded obesity and measured obesity with the first incident MOF in the observation period. The adjusted models included age, sex, prior fracture, prolonged glucocorticoid use, COPD diagnosis, alcohol/substance abuse diagnosis, rheumatoid arthritis diagnosis, and income quintile, while the unadjusted models included only the obesity covariate. The proportional hazards assumption was confirmed by including a two-way interaction term for each obesity measure with the logarithm of the follow-up duration [24]. Hazard ratios (HRs) and 95% confidence intervals (95% CIs) were produced for each model. In addition, we estimated the adjusted HRs and 95% CIs for the association of MOF and BMI categories when cohort members were classified as underweight, normal weight [referent], overweight and obese. We tested whether osteoporosis, osteoporosis treatment and sex moderated the relationship of ICD-coded obesity and measured obesity with MOF by including the following interaction terms: ICD-coded obesity*osteoporosis, measured obesity*osteoporosis, ICD-coded obesity*osteoporosis treatment, measured obesity*osteoporosis treatment, ICD-coded obesity*sex and measured obesity*sex; we tested each interaction term in a separate model. We also tested the associations of ICD-coded obesity and measured obesity with each MOF site. Given that the association between obesity and MOF may be mediated by BMD [25, 26], we fit an additional model that adjusted for femoral neck BMD.

We used the Akaike information criterion (AIC) and Bayesian information criterion (BIC) to assess model fit. Specifically, AIC and BIC statistics provided information on whether ICD-coded obesity or measured obesity resulted in a better-fitting model. In addition, area under the receiver operating characteristic curve (AUROC) and the net reclassification index (NRI) were used to test the predictive performance of ICD-coded obesity versus measured obesity. AUROC was estimated from multivariable logistic regression models. We compared the AUROC differences using the method reported by Delong et al. [27]. The NRI statistic can overcome some of the drawbacks of using AUROC analysis [28]. Ten-year probabilities for MOF associated with ICD-coded obesity and measured obesity were estimated using Cox proportional hazards models. Both category-based and category-free NRI estimates were produced. In the category-based NRI analysis, individuals with 20% or higher ten-year MOF probability were classified as high risk and all others were classified as low risk [29]. Continuous probabilities were used for category-free NRI analysis. Finally, the Hosmer-Lemeshow test [30] was used to assess calibration (i.e., observed versus predicted MOF probabilities) for models evaluating ICD-coded obesity and measured obesity. Predicted probabilities in deciles were estimated using multivariable logistic regression models. Observed probabilities were the proportion of events in each decile group. The models used for estimating AIC, BIC, AUROC, and predicted probabilities were adjusted for age, sex, prior fractures, prolonged glucocorticoid use, COPD diagnosis, alcohol/substance abuse diagnosis, rheumatoid arthritis diagnosis, and income quintiles. All analyses were performed with SAS (Version 9.4, SAS Institute Inc., Cary, NC).

## Results and discussion

After excluding ineligible individuals (Fig 1), our final cohort included 61,854 individuals aged 50 years or older, representing 86.2% of the initial cohort. Average age of the final cohort was 66.3 years. There were 2226 (3.6%) and 16249 (26.3%) individuals with ICD-coded obesity and measured obesity, respectively. The sensitivity, specificity, PPV and NPV for ICD-coded obesity using measured obesity as the reference standard were 0.11 (95% CI: 0.10, 0.11), 0.99 (95% CI: 0.99, 0.99), 0.79 (95% CI: 0.77, 0.81) and 0.76 (95% CI: 0.75, 0.76), respectively. We found similar patterns in ICD-coded obesity versus measured obesity after stratifying our analysis by osteoporosis treatment (with treatment: sensitivity 0.09 [95% CI: 0.08, 0.10]; specificity 0.99 [95% CI: 0.99, 0.99]; PPV 0.73 [95% CI: 0.69, 0.76]; NPV 0.82 [95% CI: 0.82, 0.83]; without treatment: sensitivity: 0.12 [95% CI: 0.11, 0.12]; specificity 0.99 [95% CI: 0.99, 0.99]; PPV 0.81 [95% CI: 0.79, 0.83]; NPV 0.71 [95% CI: 0.70, 0.71]) and by sex (males: sensitivity 0.10 [95% CI: 0.09, 0.12]; specificity 0.99 [95% CI: 0.99, 0.99]; PPV 0.76 [95% CI: 0.70, 0.82]; NPV 0.77 [95% CI: 0.76, 0.78]; females: sensitivity: 0.11 [95% CI: 0.10, 0.11]; specificity 0.99 [95% CI: 0.99, 0.99]; PPV 0.79 [95% CI: 0.77, 0.81]; NPV 0.76 [95% CI: 0.75, 0.76]).

Characteristics of the cohort stratified by ICD-coded obesity and measured obesity are reported in Table 1. Mean BMI was similar for ICD-coded obesity and measured obesity (34.6 versus 34.2 kg/m^2^), but there was a larger difference in mean BMI in those not having ICD-coded obesity and measured obesity (27.0 versus 24.7 kg/m^2^). Individuals with either ICD-coded obesity or measured obesity tended to be younger, with higher mean femoral neck T-score, more likely to have a COPD diagnosis but less likely to receive osteoporosis treatment.

**Table 1:** Descriptive characteristics of cohort stratified by ICD-coded obesity and by measured obesity.

Variable	ICD-Coded Obesity			Measured Obesity		
	Obese	Not Obese	*P*	Obese	Not Obese	*P*
n	2226	59,628	—	16,249	45,605	—
Age (years)	64.1 (8.4)	66.4 (9.8)	< 0.001	65.9 (9.1)	66.5 (10.0)	< 0.001
Body mass index (kg/m^2^)	34.6 (5.5)	27.0 (5.1)	< 0.001	34.2 (3.7)	24.7 (3.1)	< 0.001
Femoral neck T-score	-0.9 (1.0)	-1.3 (1.0)	< 0.001	-0.9 (1.0)	-1.5 (1.0)	< 0.001
Female (%)	91.2	90.3	0.156	90.9	90.1	0.007
Osteoporosis treatment (%)	27.0	41.7	< 0.001	29.7	45.3	< 0.001
Prior fractures (%)	9.0	7.8	0.030	7.7	7.8	0.704
Rheumatoid arthritis diagnosis (%)	2.3	3.3	0.010	3.7	3.1	0.001
Prolonged glucocorticoid use (%)	4.9	4.8	0.891	5.8	4.5	< 0.001
Alcohol/substance abuse diagnosis (%)	0.5	0.6	0.864	0.4	0.6	0.011
COPD diagnosis (%)	6.6	4.9	< 0.001	5.3	4.9	0.028
Income quintile (%)						
1 (lowest)	16.3	16.1	0.350	17.5	15.6	< 0.001
2	22.0	20.7		22.0	20.3	
3	23.2	22.3		23.4	21.9	
4	17.8	19.0		18.2	19.2	
2	20.1	21.1		18.1	22.2	
missing	0.7	0.7		0.7	0.8	

Unless otherwise specified, continuous variables are shown as means (standard deviations). COPD: Chronic obstructive pulmonary disease.

**Fig 2:**
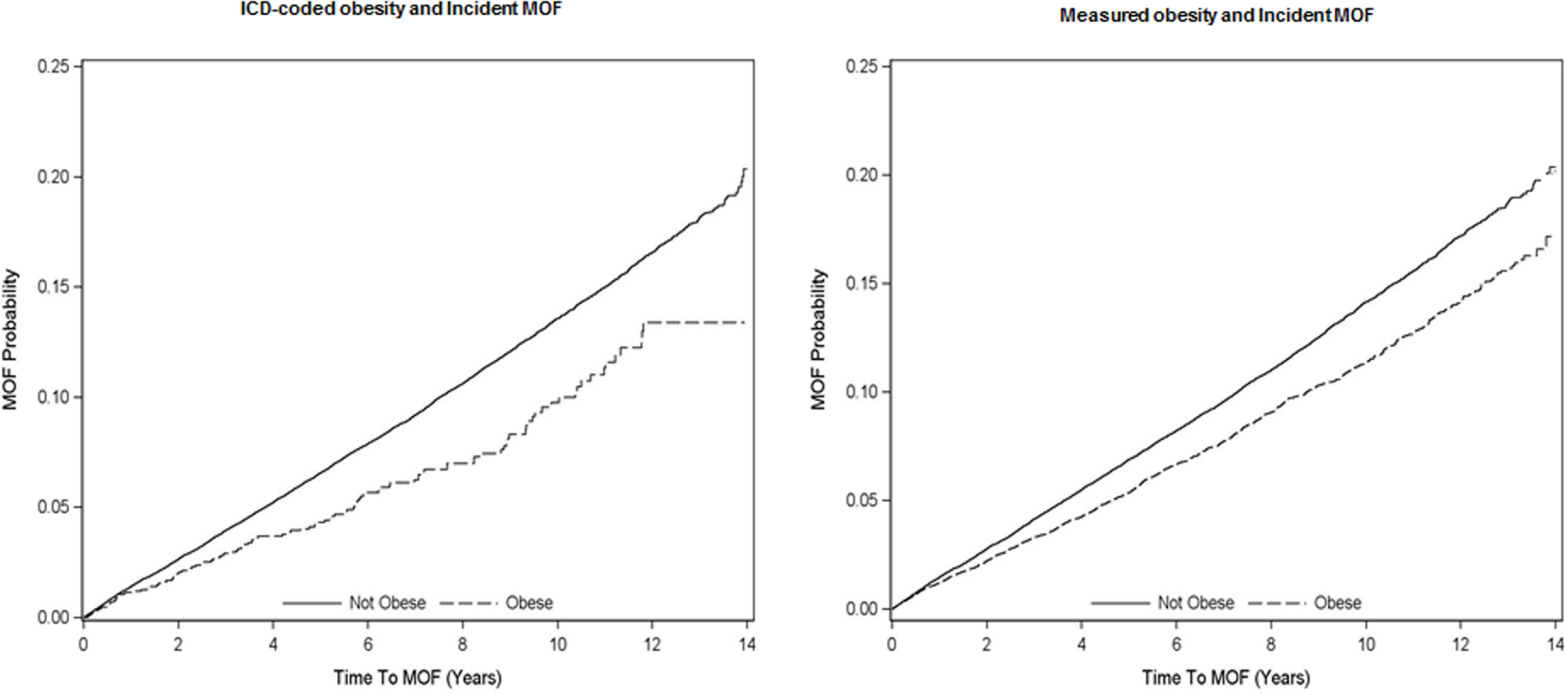
Cummulative incidence curves for major osteoporotic fracture (MOF) associated with ICD-coded obesity versus no ICD-coded obesity (left panel) and measured obesity versus no measured obesity (right panel).

During an average of 6.7 years (max: 14.0 years) of follow-up, we identified 5972(9.7%) individuals with incident MOF, including 1836 (3.0%) hip fractures, 2271 (3.7%) forearm fractures, 1379 (2.2%) clinical spine fractures and 1235 (2.0%) humerus fractures. Individuals with ICD-coded obesity had a lower proportion of MOF than those without ICD-coded obesity (5.6% versus 9.8%; *P*< 0.001) with similar lower MOF rates for individuals with measured obesity (7.8% versus 10.3%, respectively; *P*< 0.001). The cumulative incidence of MOF was lower in individuals who were obese compared to individuals who were not obese regardless of how obesity was defined (log-rank test *P* < 0.001 for ICD-coded obesity; log-rank test *P* < 0.001 for measured obesity; Fig 2). In the unadjusted models, ICD-coded obesity (HR 0.71; 95% CI: 0.59, 0.85; Table 2) and measured obesity (HR 0.81; 95% CI: 0.76, 0.86) were both associated with decreased risk of incident MOF, and these relationships remained statistically significant after adjusting for the non-BMD covariates. ICD-coded obesity and measured obesity were no longer significantly associated with MOF after further adjusting for femoral neck BMD. The association of ICD-coded obesity and measured obesity with MOF was not moderated by osteoporosis, osteoporosis treatment or sex (all *P* for interactions > 0.05). With normal weight as the referent category for BMI, underweight was significantly associated with increased MOF risk (adjusted HR 1.55; 95% CI: 1.34, 1.81), whereas overweight (adjusted HR 0.83; 95% CI: 0.79, 0.89) and obese (adjusted HR 0.77; 95% CI: 0.72, 0.82) were associated with decreased MOF risk (S1 Table). Measured obesity was significantly associated with reduced risk for hip (adjusted HR 0.71; 95% CI: 0.62, 0.80) and forearm fracture (adjusted HR 0.86; 95% CI: 0.77, 0.95), but with increased risk for humerus fracture (adjusted HR 1.26; 95% CI: 1.11, 1.43). ICD-coded obesity was not significantly associated with individual fracture sites (S2 Table).

**Table 2:** Hazard ratios (HRs) and 95% confidence intervals (95% CIs) for incident major osteoporotic fracture (MOF) associated with ICD-coded obesity and measured obesity.

Independent Variable	Model	HR (95% CI)
ICD-coded obesity	Unadjusted	**0.71 (0.59, 0.85)**
	Adjusted^a^	**0.83 (0.70, 0.99)**
	Adjusted^a^ + femoral neck BMD	0.97 (0.81, 1.16)
Measured obesity	Unadjusted	**0.81 (0.76, 0.86)**
	Adjusted^a^	**0.83 (0.78, 0.88)**
	Adjusted^a^ + femoral neck BMD	1.04 (0.97, 1.11)

^a^Adjusted for age, sex, prior fractures, prolonged glucocorticoid use, COPD diagnosis, alcohol/substance abuse diagnosis, rheumatoid arthritis diagnosis, and income quintiles.

NA: Not applicable. Bold-faced values indicate statistical significance at α = 0.05.

In the adjusted models, the AUROC estimates for incident MOF were 0.648 (95% CI: 0.640, 0.655) for ICD-coded obesity and 0.650 (95% CI: 0.642, 0.657) for measured obesity, respectively (*P* = 0.056 for AUROC difference). ICD-coded obesity resulted in higher AIC (122954 versus 121960) and BIC values (123041 versus 122054) than measured obesity for predicting MOF risk (Table 3), indicating worse model fit. The overall category-based NRI for ICD-coded obesity versus measured obesity was -0.01 (95% CI: -0.01, -0.002; Table 4). The NRI statistics for MOF events were negative (NRI -0.01; 95% CI: -0.01, -0.004), and for non-events were positive (0.002; 95% CI: 0.001, 0.003). In the category-free NRI analysis, the overall NRI, the NRI for MOF events, and the NRI for non-events were -0.08 (95% CI: -0.11, -0.06), -0.61 (95% CI: -0.63, -0.59), 0.52 (95% CI: 0.52, 0.53), respectively. Using the Hosmer-Lemeshow test, there was no evidence of statistically significant miscalibration in observed versus predicted MOF probabilities for models evaluating ICD-coded obesity (*P* = 0.162) or measured obesity (*P* = 0.232).

**Table 3:** Akaike information criterion (AIC) and Bayesian information criterion (BIC) for incident major osteoporotic fracture (MOF) associated with ICD-coded obesity and measured obesity.

Independent Variable	AIC	BIC
ICD-coded obesity	122954	123041
Measured obesity	121960	122054

All values were adjusted for age, sex, prior fractures, prolonged glucocorticoid use, COPD diagnosis, alcohol/substance abuse diagnosis, rheumatoid arthritis diagnosis, and income quintiles.

**Table 4:** Net reclassification indices (NRIs) and 95% confidence intervals (95%CIs) for incident major osteoporotic fracture (MOF) associated with ICD-coded obesity and measured obesity.

Independent Variable	NRI Type	For Event	For Non-event	Overall
ICD-coded obesity versus measured obesity	Category-based	**-0.01 (-0.01, -0.004)**	**0.002 (0.001, 0.003)**	**-0.01 (-0.01, -0.002)**
	Category-free	**-0.61 (-0.63, -0.59)**	**0.52 (0.52, 0.53)**	**-0.08 (-0.11, -0.06)**

NA: Not applicable. Bold-faced values indicate statistically significant at *α* = 0.05.

In this historical cohort study, we confirmed that ICD-coded obesity and measured obesity were significantly associated with reduced incident MOF risk; risk estimates for incident MOF obtained using ICD-coded obesity and measured obesity were identical. These results suggest that ICD-coded obesity may help to stratify risk of MOF when data on measured obesity are not available, despite the low sensitivity of ICD-coded obesity from administrative data.

The low sensitivity of ICD-code obesity has also been reported in other studies [7, 8]. In administrative databases, ICD-coded obesity is more likely to be missed in obese individuals with less marked elevations in BMI values or without obesity-related complications [7, 8]. A better obesity reporting system (i.e., incentivizing physicians for diagnosis and coding of obesity in clinical practice; use of BMI data from other resources such as electronic health records) may help to resolve this problem.

To the best of our knowledge, this is the first study showing the utility of ICD-coded obesity in determining fracture risk. Consistent with previous studies [25, 26], the effects of obesity on MOF, regardless of whether it is defined by diagnosis codes or by measured body weight and height, are largely explained by higher BMD. Measured obesity was associated with reduced fracture risk overall, but with increased fracture risk at the humerus site, again consistent with previous studies [9, 31]. Despite the overall protective relationship between obesity and fracture, it should be noted that the relationship between obesity and fracture is still controversial [9].

The protective relationship between obesity and MOF reflects several potential mechanisms. Individuals with obesity have greater mechanical loading on the bone, which stimulates bone formation and increases BMD [32]. In addition, the influence of fat on bone involves interrelated regulatory pathways [33]. For example, fat tissue is involved in the metabolism of bone-active hormones, such as estrogen and leptin, which can have a stimulatory effect on bone formation [34, 35]. Besides the direct effect of obesity on BMD, obese individuals typically have more soft tissue padding than non-obese individuals. This could contribute to impact force attenuation and lower fracture risk in individuals with obesity [36].

Our study has some limitations. This research was primarily conducted based on a clinical registry database for DXA scans, and this may limit the generalizability of our results to other populations. However, the prevalence of measured obesity in our study is consistent with the prevalence in the Manitoba population [37]. This indicates that our clinical registry is representative of the general Manitoba population. In addition, our study could not test for potential interactions by race / ethnicity as the vast majority (about 99%) of our study population was Caucasian.

## Conclusions

We have demonstrated the protective relationship between ICD-coded obesity and osteoporotic fractures. Further studies are warranted to determine the utility of ICD-coded obesity for determining risk for conditions other than osteoporotic fracture, such as such as cardiovascular diseases and diabetes.

## Supporting information

S1 Table Hazard ratios (HRs) and 95% confidence intervals (95% CIs) for the association of major osteoporotic fracture (MOF) and BMI categories when cohort members were classified as underweight, normal weight [referent], overweight and obese. ^a^Adjusted for age, sex, prior fractures, prolonged glucocorticoid use, COPD diagnosis, alcohol/substance abuse diagnosis, rheumatoid arthritis diagnosis, and income quintiles. Bold-faced values indicate statistical significance at α = 0.05.
(PDF)

S2 Table Hazard ratios (HRs) and 95% confidence intervals (95% CIs) for the association of each major osteoporotic fracture (MOF) site with ICD-coded obesity and measured obesity. HRs were adjusted for age, sex, prior fractures, prolonged glucocorticoid use, COPD diagnosis, alcohol/substance abuse diagnosis, rheumatoid arthritis diagnosis, and income quintiles. Bold-faced values indicate statistical significance at α = 0.05.
(PDF)
