# Supplementary material for: International Classification of Diseases (ICD)-coded obesity predicts risk of incident osteoporotic fracture
Source: PLoS One. 2017 Dec 7;12(12):e0189168. doi: 10.1371/journal.pone.0189168 (PMC5720696; doi:10.1371/journal.pone.0189168)
Supplement: S1 Table — aAdjusted for age, sex, prior fractures, prolonged glucocorticoid use, COPD diagnosis, alcohol/substance abuse diagnosis, rheumatoid arthritis diagnosis, and income quintiles. Bold-faced values indicate statistical significance at α = 0.05. (PDF) [file pone.0189168.s001.pdf]

**Model**	**Underweight**	**Normal weight**	**Overweight**	**Obese**
Unadjusted	**1.69 (1.45, 1.96)**	1 (ref)	**0.86 (0.81, 0.91)**	**0.77 (0.72, 0.82)**
Adjusted[a]	**1.55 (1.34, 1.81)**	1 (ref)	**0.83 (0.79, 0.89)**	**0.77 (0.72, 0.82)**
Adjusted[a] + femoral neck BMD	1.14 (0.98, 1.33)	1 (ref)	0.98 (0.92, 1.04)	1.03 (0.96, 1.10)
