# Supplementary material for: International Classification of Diseases (ICD)-coded obesity predicts risk of incident osteoporotic fracture
Source: PLoS One. 2017 Dec 7;12(12):e0189168. doi: 10.1371/journal.pone.0189168 (PMC5720696; doi:10.1371/journal.pone.0189168)
Supplement: S2 Table — HRs were adjusted for age, sex, prior fractures, prolonged glucocorticoid use, COPD diagnosis, alcohol/substance abuse diagnosis, rheumatoid arthritis diagnosis, and income quintiles. Bold-faced values indicate statistical significance at α = 0.05. (PDF) [file pone.0189168.s002.pdf]

Independent Variable	Hip	Forearm	Clinical Spine	Humerus
ICD-coded obesity	0.86 (0.58, 1.26)	1.03 (0.79, 1.35)	0.95 (0.64, 1.42)	1.28 (0.91, 1.80)
Measured obesity	**0.71 (0.62, 0.80)**	**0.86 (0.77, 0.95)**	0.98 (0.87, 1.12)	**1.26 (1.11, 1.43)**
